# Supplementary material for: Loss of H3K9 trimethylation leads to premature aging
Source: Res Sq. 2024 Dec 16:rs.3.rs-4012025. Preprint. [Version 1] doi: 10.21203/rs.3.rs-4012025/v1 (PMC11702797; doi:10.21203/rs.3.rs-4012025/v1)
Supplement: Supplement 1 [file NIHPPRS4012025v1-supplement-1.pdf]

Supplementary Table 1.

Table showing the category of the top 50 significantly changed TE transcripts based on *p*-value in spleen, small intestine, skin, skeletal muscle, liver, kidney and liver from young and old WT mice

Supplementary Files

This is a list of supplementary files associated with this preprint. Click to download.

- MrabtietalSupplementaryTable1.xlsx
